# Systematic review of resilience-enhancing, universal, primary school-based mental health promotion programs

**DOI:** 10.1186/s40359-018-0242-3

**Published:** 2018-07-05

**Authors:** Amanda Fenwick-Smith, Emma E. Dahlberg, Sandra C. Thompson

**Affiliations:** Western Australian Centre for Rural Health, University of Western Australia, 167 Fitzgerald St, Geraldton, WA 6531 Australia

**Keywords:** Mental health, Health promotion, Primary school, Resilience, Universal intervention, Child

## Abstract

**Background:**

Wellbeing and resilience are essential in preventing and reducing the severity of mental health problems. Equipping children with coping skills and protective behavior can help them react positively to change and obstacles in life, allowing greater mental, social and academic success. This systematic review studies the implementation and evaluation of universal, resilience-focused mental health promotion programs based in primary schools.

**Methods:**

A systematic review of literature used five primary databases: PsycINFO; Web of Science; PubMed; Medline; Embase and The Cochrane Library; and keywords related to (a) health education, health promotion, mental health, mental health promotion, social and emotional wellbeing; (b) school health service, student, schools, whole-school; (c) adolescent, child, school child, pre-adolescent; (d) emotional intelligence, coping behavior, emotional adjustment, resilienc*, problem solving, to identify relevant articles. Articles included featured programs that were universally implemented in a primary school setting and focused on teaching of skills, including coping skills, help-seeking behaviors, stress management, and mindfulness, and were aimed at the overall goal of increasing resilience among students.

**Results:**

Of 3087 peer-reviewed articles initially identified, 475 articles were further evaluated with 11 reports on evaluations of 7 school-based mental health promotion programs meeting the inclusion criteria. Evaluation tools used in program evaluation are also reviewed, with successful trends in evaluations discussed. Encouraging results were seen when the program was delivered by teachers within the schools. Length of programing did not seem important to outcomes. Across all 7 programs, few long-term sustained effects were recorded following program completion.

**Conclusions:**

This review provides evidence that mental health promotion programs that focus on resilience and coping skills have positive impacts on the students’ ability to manage daily stressors.

## Background

This review looks at resilience-boosting mental health promotion programs implemented universally at schools for primary school-aged children (5–12 years). Wellbeing and resilience are important in preventing and reducing the severity of mental health problems. The skills of problem solving, building and maintaining interpersonal relationships, and realistic goal-setting are well-established as enhancing an individual’s ability to contribute meaningfully in daily life. There is substantial literature on resilience [1] which is defined as a capacity or set of skills that allows a person to “prevent, minimize or overcome the damaging effects of adversity” [2] and includes factors that are internal and external to the person - emotions, behavior, biology, development, and context affect mental health [3]. Potential risks for poor self-esteem and mental health can be overcome by protective factors, including one’s coping skills, healthy family and social relationships, help-seeking behaviors, and meaningful activities in interactions [4].

Resilience theory states that all children, regardless of risk or current mental health status, can benefit from help and support in the development of effective, mentally-healthy strategies and resilience skills [5]. Support for and a focus on the development of children’s resilience skills does not lead to a risk-free life, but can increase a child’s ability to seek support while building their self-worth and self-efficacy. By providing children with skills with which to cope with negative life stressors through the promotion of resilience and protective factors, children can thrive despite obstacles [6]. An argument for a population approach for mental health strengthening can be extrapolated from Geoffrey Rose’s argument that the largest number of cases of ill health happen not in those at high risk, but in those who have just some risk, simply because in a normal population distribution more people (and hence adverse events) will occur to them [7]. Since all people experience adversity at some point in their life, teaching strategies for resilient thinking would be better applied in advance to the potential “at risk” population. The positive outcomes and possibilities associated with strengthening children’s resilience universally applied can act as a mitigating approach, allowing for early support and strengthening of mental health, rather than requiring interventions for acute situations in the future [8]. The approach of boosting resilience can enhance children’s abilities to self-protect, as well as being an effective counter to offset the effects of maltreatment and potential traumatic life events [9–11]. As such, universal application of programs to enhance resilience stands as not only useful for those recognized as being *at risk* and who require additional mental health support currently, but also as a protective shield for all children moving through life.

### Universal, school-based programing

School-based mental health promotion programs delivered to all students within a class, grade, or the entire school are categorized as universal programs. In developed countries, all children are required to attend school, making it an ideal setting for programs providing key interventions for children, particularly children from challenged families, homes and communities that may not have easy access to community or home-based intervention programs [12]. Mental health promotion programs have been developed and implemented in schools using a variety of different approaches. Many mental illness prevention or intervention programs use a targeted approach, focusing on children deemed at risk due to their background, history or signs of mental health problems, usually based upon defined socio-demographic factors or certain behavioral characteristics.

Universal programs vary in their approach and implementation. Some universal programs are class-based, with weekly sessions delivered by classroom teachers or program staff to the entire classroom. Another universal approach is to change the entire school environment to be friendlier and more supportive of positive mental health messages, and this is often implemented in combination with class-based approaches [13]. Class-based, universal mental health promotion programs vary in their aims, focusing on different elements of cognitive or affective skills and behaviors, environmental or cultural factors, while increasing knowledge of mental health and resources.

Mental health promotion programs specifically targeting resilience may be referred to as social and emotional learning programs, mindfulness programs, stress management programs, or emotional wellbeing programs and vary in terms of curriculum, length and implementation, and use of different tools and activities to convey key themes and topics. Methods of delivery vary as well, including the use of clinical tools, educational resources, training of teachers and parents, changes to school systems and resources, and use of narrative tools. As such, the curricula used in these programs vary, although all utilize a pre-established definition of resilience and the desired outcomes to be achieved from a social and emotional learning program. The most effective social and emotional learning curricula are highly interactive and use a variety of educational tools, addressing both specific and general skills, and are delivered in supportive environments [14]. Mental health promotion programs promoting resilience focus on the development of coping skills, mindfulness, emotion recognition and management, empathic relationships, self-awareness and efficacy, and help-seeking behavior. Secondary outcomes often include decreased symptoms of anxiety, depression, and increased academic outcomes.

### Relevant research reviews

Given the importance and reach of school settings, many reports describe universal, school-based mental health promotion programs. Prior reviews have explored school-based mental health promotion programs in different contexts, countries, applications, and within specific demographic parameters. There are many reviews addressing targeted programs aimed at suicide prevention, sexual health, substance abuse and misuse, physical activity and nutrition improvement and these often measure as secondary outcomes changes in self-efficacy, coping and resilience skills [5, 15–17]. A number of reviews analyzing mental health promotion programs that focus on resilience across a range of age groups have established that school-based interventions can have significant impacts on achievement, social and emotional skills, behavior, and symptoms of anxiety and depressive disorders [12, 16, 18]. In their 2017 review, Dray and colleagues looked at control-based trial evaluations of programs of universal resilience-programing in schools spanning all ages, reporting on those that yielded significant results in resilience factor changes. Durlak and colleagues compared 213 programs, also targeting all age groups, assessing the outcomes on attitudes, behaviors and academic performance and analyzing effect size and factors that moderate program outcomes. Waere and Mind assessed the key features that make school-based curricula successful as an approach, highlighting the importance of social and emotional competence as part of the curriculum within schools [12]. Another review considered studies on mental health promotion programs solely conducted with control and comparison groups [19].

### The current review

This review aims to inform policy, programing and evaluation of universal, resilience-focused mental health interventions for primary school-aged children as it focuses on the specific tools and key elements for the population that will benefit the most from increased resilience in an easy-to-reach setting, aspects which have not been highlighted in previous reviews. The multitude of existing mental health promotion programs highlights the need to establish what specific elements and evaluations contribute to successful programing. Unlike previous reviews, this review focuses on programs delivered solely to primary school students (aged 5–12 years), as there is evidence that the younger the implementation of mental health promotion and resilience programing, the greater the positive effect [3, 20, 21]. Rather than focusing on the program curriculum, it considers the criteria for implementation and key elements of programing for a comprehensive intervention, highlighting the elements of that allow for best program fidelity and student engagement. It also describes the criteria and outcome measures (tools and methods) used in implementing and evaluating resilience-focused, universal school-based mental health promotion programs.

## Methods

Studies eligible for inclusion were published from 2002 to 2017, describe mental health promotion programs focusing on resilience and protective factors, and were delivered universally at schools for primary school children aged 5–12 years. A universal program is defined as being a program offered for a specific all-inclusive group, whether it be the entire school, grade or classroom. All students within the group participate in at least one component of the program, regardless of their mental health status and risk factors. Resilience is defined as a capacity or set of skills that allows a person to “prevent, minimize or overcome the damaging effects of adversity” [2], through the promotion of protective factors including coping skills, peer socialization and empathy building, self-efficacy, help-seeking behaviors, mindfulness and emotion literacy.

### Search procedures

A preliminary review of literature revealed key terms related to resilience-focused, school-based, universal mental health promotion programs. A broad search strategy was then developed to identify relevant peer-reviewed articles in five primary databases: PsycINFO; Web of Science; PubMed; Medline; Embase and The Cochrane Library. The search strategy was modified as necessary for advanced searches of each database, using keyword search criteria: (a) health education, health promotion, mental health, mental health promotion, social and emotional wellbeing; (b) school health service, student, schools, whole-school; (c) adolescen*, child, school child, pre-adolescent; (d) emotional intelligence, coping behavior, emotional adjustment, resilienc*, problem solving. Searches were conducted in September 2016 and updated in May 2018. Articles were initially screened by abstract by the lead author. All full-text articles were reviewed by two reviewers, with additional checks and consultations with other authors, to ensure consensus around those articles where eligibility was less clear. Snowball citation was used to identify other relevant articles.

#### Inclusion criteria

To be included in the review, each study had to meet the following criteria: (a) adhere to the above definition of a universal program; (b) be based in a primary school; (c) be delivered to children aged between 5 and 12 years of age; (d) focus on resilience and protective factors (meeting the above definition); (e) contain a qualitative, quantitative or mixed-methods evaluation of the program; (f) be published in English since 2002 in a peer-reviewed journal.

#### Exclusion criteria

Programs targeting specific behaviors where resilience is a secondary outcome, or programs primarily focusing on post-traumatic stress among students affected by natural disasters or war were not included**.** Programs with the ultimate goal and outcome measurements relating to a specific behavior, emotional condition or mental illness were not included, even if the tools taught in the intervention could be classified as resilience promoting. Universal programs that sought to change school atmosphere through teacher resilience training, or increasing school health services were not included. After-school or recess resilience programing was not included, even if it took place at a school. Programs that were available but not implemented universally were not included, as the self-selecting nature of optional programing is unlikely to reach the most at-risk children, and such programs do not insure a comprehensive program for all students regardless of risk. Studies where many students were outside of the age group and during a transition period between different schools were not included. Resilience programing that fits our inclusion criteria but is solely delivered to a population that has been exposed to high stress situations and is at risk or may develop PTSD are not included. Unpublished dissertations, grey literature and reports were not included.

#### Excluded studies

It is worth commenting upon how exclusion criteria were applied in practice. A number of programs were not included in this review despite having a resilience focus, being universally-delivered and school-based because they have not been reported upon within the preceding 15 years (since 2002). Other excluded programs had an ultimate goal that was not general mental health promotion program, but rather aimed at addressing a specific condition or behavior through the promotion of certain resilience skills and protective factors. Notable programs include the Penn Resilience Program, which has been shown to reduce depressive symptoms through the cognitive-behavioral therapy programing, including the promotion of coping skills [22]. The Good Behavior Games specifically target behavior control through the promotion of resilience, but fall outside of the age range of this review [23]. REACH for RESILIENCE promotes resilience skills to prevent anxiety problems, and targets very young children [24]. The nation-wide Australian program, beyondblue, focuses on social and resilience skills to prevent depression, targeting adolescents [25]. Evaluations of the FRIENDS program were not included as it targets childhood anxiety through the promotion of social-emotional skills [26]. Another exclusion was the Aussie Optimism: Positive Thinking Skills Program (AOP-PTS) which promotes social and coping skills to prevent and address depression symptoms [27].

#### Article quality assessment

The Mixed Methods Appraisal Tool (MMAT) was used to assess the quality of included studies and provides a validated method of assessing qualitative, quantitative, and mixed methods studies. After the initial screening, articles were scored based on the criteria for each respective study [28]. Two researchers independently assessed each article [29]. Of note, the tool does not address the quality of the reporting, but only the quality of the reported methods of the study.

## Results

**Fig. 1:**
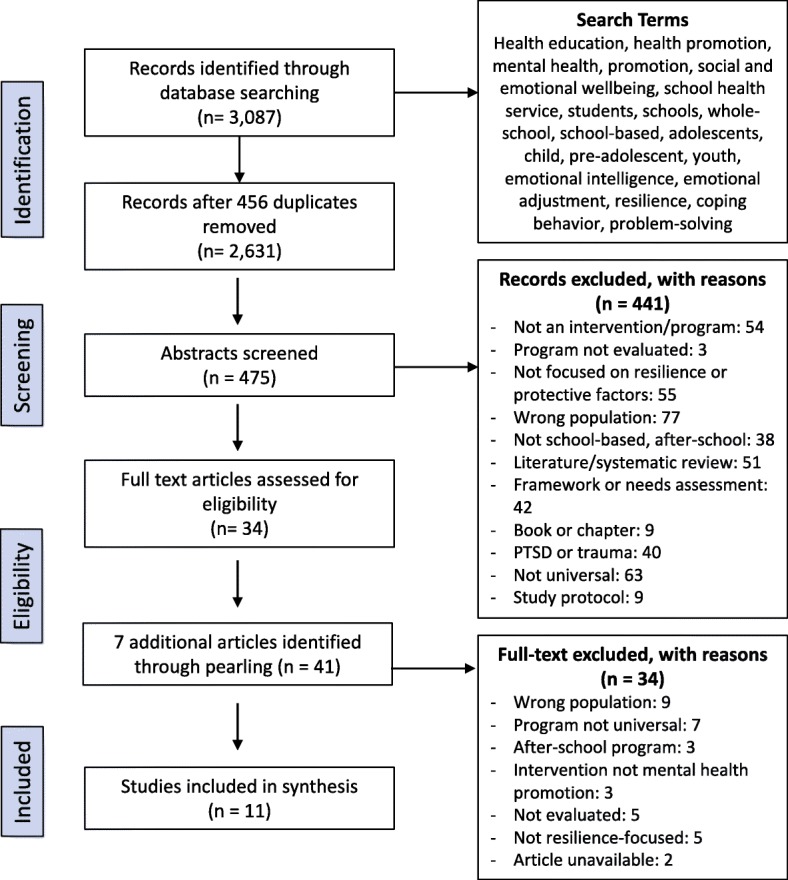
Flow diagram of selection process for relevant literature

The initial search strategy shows that of an initial 3087 publications identified using the search terms and following abstract assessment of 475 references, 34 articles were selected for full-text assessment. An additional 7 articles were identified through citation snowballing and after reading of the full-text so that 41 articles were fully assessed for eligibility. A total of 11 studies reporting on 7 programs met all the inclusion criteria (Fig. 1), with key characteristics including MMAT scores recorded (Table 1). The most common reasons for exclusion were: focus on trauma, incorrect age group or target population; not meeting our definition of universal programs; and lack of focus on resilience and protective factors. Included articles. Key elements of each program’s curriculum and implementation are shown (Table 2).

**Table 1:** Summary of Articles Included in the Review

First author, year published Study type	Program Name	Location	Study Type	Sample Size	Aim of Program and Study	MMAT Score
Malti (2008)[36] Program Evaluation: Relationships as key to student development	RALLY	United States	Quasi-experimental, Mixed methods	92 students	- Improved resilience outcomes, learning interest and decrease risk-taking. - Assess program implementation quality	100%
Sibinga (2016)[30] School-Based Mindfulness Instruction: An RCT	Mindfulness-Based Stress Reduction (MBSR)	United States (Baltimore, Maryland)	Randomized, Active Controlled Trial	Interv: 159 students	- Improve psychological functioning to decrease negative effects of stress - Reduce worries about future	50%
Kraag (2009)[39] “Learn Young, Learn Fair”, a stress management program for fifth and sixth graders: longitudinal results from an experimental study	Learn Young, Learn Fair	Netherlands	Cluster Randomized Controlled Trial	Interv: 693 students (26 schools) Control: 732 students (24 schools)	- Improve stress management and coping skills - Reduce anxiety and depression symptoms and incidence	100%
Mishara (2006)[32] Effectiveness of a mental health promotion program to improve coping skills in young children: Zippy’s Friends	Zippy’s Friends	Denmark & Lithuania	Non-randomized Experimental Trial	Students Lithuania: Interv: 314 Control: 104 Denmark: Interv: 322 Control: 110	- Increase ability to cope with everyday life adversities and negative events - Decrease problems that arise from stressful situations - Development of adaptive coping skills	75%
Clarke (2014)[33] Evaluating the implementation of a school-based emotional well-being program: a cluster randomized controlled trial of Zippy’s Friends for children in disadvantaged primary schools	Zippy’s Friends	Ireland	Cluster Randomized Controlled Trial	Interv: 544 students Control: 222 students	- Increase ability to cope with everyday life adversities and negative events - Decrease problems that arise from stressful situations - Development of adaptive coping skills	25%
Dufour (2011)[34] Improving Children’s Adaptation: New Evidence Regarding the Effectiveness of Zippy’s Friends, a School Mental Health Promotion Program	Zippy’s Friends	Canada (Quebec)	Cluster Randomized Controlled Trial	Interv: 310 students (16 classes) Control: 303 students (19 classes)	- Increase ability to cope with everyday life adversities and negative events - Decrease problems that arise from stressful situations - Development of adaptive coping skills	50%
Holen (2012)[35] The effectiveness of a universal school-based program on coping and mental health: a randomized, controlled study of Zippy’s Friends	Zippy’s Friends	Norway	Randomized Controlled Trial	Interv: 686 students (47 classes, 18 schools) Control: 638 students (44 classes, 17 schools)	- Increase ability to cope with everyday life adversities and negative events - Decrease problems that arise from stressful situations - Development of adaptive coping skills	75%
Clarke (2015)[31] Evaluating the implementation of an emotional wellbeing program for primary school children using participatory methods	Zippy’s Friends	Ireland	Participatory Workshop of Randomized Controlled Trial	Interv: 544 students Control: 222 students Workshop:	- Increase ability to cope with everyday life adversities and negative events - Decrease problems that arise from stressful situations - Development of adaptive coping skills	100%
Nielsen (2015)[37] Promotion of social and emotional competence: Experiences from a mental health intervention applying a whole school approach	Up	Denmark	Multi-component Intervention, No Control Group	589 students (2 schools)	- Enhance social and emotional competencies to improve mental health - Increase positivity of school mental health environment	50%
Caldarella (2009)[40] Promoting Social and Emotional Learning in Second Grade Students: A Study of the Strong Start Curriculum	Strong Start	United States (Utah)	Quasi-Experimental, Non-Equivalent Control Group	26 students	- Prevent future emotional and behavioral problems via the promotion of social and emotional wellbeing	50%
Yamamoto (2017) [38] Effects of the cognitive behavioral You Can Do It! Education program on the resilience of Japanese elementary school students: A preliminary investigation	You Can Do It! Education	Tokyo	Quasi-Experimental, Intervention, Control Group	125 students, intervention *n* = 78, control group =47	- Evaluate a mental health promotion program’s efficacy in enhancing resilience in schools	100%

**Table 2:** Key elements of programs reported in included studies

Program First Author (year published)	Summary	Solely class-based	Changes during delivery	Program Support	Delivered by teacher	Delivered by outsider	Significant Implementation	Age Appropriate
RALLY Malti, (2008) [36]	Multi-component program with a few components delivered universally in the classroom *Duration: school year*		✓			✓		✓
MBSR Sibinga (2016) [30]	Based on adult mindfulness curriculum, three core sections focusing on didactic mindfulness, mindfulness practice, applications to life *Duration: 12 weeks*	✓				✓		✓
Learn Young, Learn Fair Kraag (2009) [39]	Weekly hour-long lessons with optional, additional five weekly booster sessions, homework assignments, daily exercises *Duration: 7 months*	✓		✓	✓		✓	
Zippy’s Friends Mishara (2006) [32] Clarke (2014) [33] Dufour (2011) [34] Holen*.* (2012) [35] Clarke (2015) [31]	24 sessions conducted each week built around 6 stories of a group of children and their pet insect Zippy; each module focusing on a theme with participatory activities *Duration*: *24 weeks*	✓	✓	✓	✓		✓	✓
Up Nielsen (2015) [37]	Year-long program with four themes focusing on education and activities for school children, staff skill development, parental involvement and school initiatives *Duration: 1 year*			✓		✓		
Strong Start Caldarella (2009) [40]	Programing with weekly direct instruction sessions with scenarios, role plays, think/pair/share activities, children’s literature and a curriculum mascot *Duration: 6 weeks*	✓	✓		✓		✓	✓
You Can Do It! Education Yamamoto (2017) [38]	8 × 45 min intervention sessions focused on themes such as emotions, ‘resilience boosters’, and ‘using your head’ accompanied by activities that promote the topic and foster resilience and emotional intelligence *Duration 20 weeks (program delivery was affected by time constraints in the school, school vacations, and classroom obligations. Hence, a reduced smaller number of sessions were conducted during the time allotment of 20 weeks.)*	✓	✓			✓		✓

### Aim of the program

The aims of the seven programs (reported on in eleven articles) included varied in their approach to resilience and the protective factors they sought to address. All six programs sought to increase social and emotional competencies with the ultimate aim of increasing mental wellbeing and future protection from risks. Six articles, addressing 2 different programs, Mindfulness-Based Stress Reduction and Zippy’s Friends, specifically sought to improve psychological functioning with the goal of ameliorating the negative effects of stress and increasing coping skills [30–35]. The RALLY program aimed at increasing the prevalence of resilience protective factors in students, with a particular focus on academic outcomes and learning potential [36] while the Up program, a social and emotional competencies program, aimed at enhancing existing competencies and decreasing inequity in social and emotional competencies across socioeconomic lines [37]. The You Can Do It! (YCDI!) Education program sought to ameliorate children’s ability to positively control their emotions in daily life [38]. All programs sought to improve the outcomes of one or more protective factors, hypothesizing increased resilience as a result. A strong emphasis on increased coping skills and strategies as well as improved relationships was evident in all the programs.

### Target population

Universal programs demand the application of the program to an entire cohort of students, but *how* that was done varied from delivering the program to an entire class, across an entire grade or across multiple schools. As such, sample size varied significantly between studies. Details of sample populations (Table 1) show all but two studies were implemented and evaluated across multiple schools, with ten of eleven conducted across multiple classrooms [30–35, 37–39]. Age groups varied across the programs, with 4 studies addressing populations 10 years and above [30, 36, 38, 39], and 6 studies addressing populations younger than 10 years of age [31–35, 40]. Socio-demographic profiles of students varied across studies. Four studies described programs delivered at socio-economically disadvantaged schools [30, 31, 33, 36] whereas four programs took place in middle or upper class neighborhoods [32, 37, 39, 40]. Dufour et al*.* (2011) did not report on socio-demographic data of students who received the program [34] whereas the students involved in the report by Holen *et al* (2012) were from homes where parents had educational attainment levels higher than the national average [35]. Yamamoto et al. (2017) delivered the program to students in the Tokyo Metropolitan Area, making no demographic distinctions, other than to address the specific contextual implications of Japanese emotion- and stress-culture as impactful in their student population [38].

### Key elements of programs

Key elements of the programs (Table 2) show that Malti et al (2008) was the only study in which the program comprised more than one student-focused component [36]. Although only a few components were delivered universally, all students were exposed to at least one component of the program [36]. The Up program included parent and teacher training, and school environment programing [37] and program fidelity and adaptability were identified as key contributing factors to successful implementation with four studies reporting high levels of program fidelity and program support [32, 33, 39, 40]. The five studies that implemented and evaluated the Zippy’s Friends program described no changes in curriculum or delivery, but allowed for activity adaptability during sessions [31–35]. Teachers delivering it felt equipped to adapt the program as they saw necessary to their class while still maintaining high program fidelity [34]. Adaptability was also highlighted as being an important program factor for the You Can Do It! Education program in Japan, where program staff translated and altered the internationally-implemented program with Japan-specific illustrations, examples and exercises to optimize the connection with students [38]. Three studies identified problems with implementation of programming due to teacher perceptions, time constraints, participation rates and class literacy levels [32, 33, 38, 40].

### Evaluation frameworks, tools and indicators

Study evaluation frameworks and indicators (summarised in Table 3) are reported with more detail on evaluation tools and methods used for evaluating elements of programing reported in Appendix. Studies varied greatly on the timing and purpose of their evaluation although all applied a combination of pre-assessment, post-assessment, process evaluation, implementation evaluation and follow up assessments. Within specific programs, different evaluations were used for different implementations and contexts. The five articles reporting on the Zippy’s Friends program utilized different evaluation methods [31–35]. Mishara and Ystgaard (2006) evaluated the implementation of Zippy’s Friends in two countries with similar socio-demographic characteristics, Lithuania and Denmark, and found similar results in outcomes of students in the intervention groups in both countries. Yamamoto et al. used a semi-experimental design with intervention and control groups and utilized three self-report scales to evaluate students [38]. Clarke evaluated a randomized-controlled trial implementation of Zippy’s Friends in Ireland using both standard measures [33] and a participatory workshop with a subsample of students. The workshop was semi-structured around three key themes: lived experiences and coping reactions; emotion recognition and regulation; and program evaluation [31]. In all articles meeting out inclusion criteria, multiple standardized, validated tools were used for evaluation measures, most commonly the Children’s Depression Inventory (CDI, Short or Complete Form) [30, 39], the Strengths and Difficulties Questionnaire [33, 35], the Schoolagers’ Coping Strategies Inventory [32, 34], and a Program Fidelity Checklist [33, 40]. Evaluation methods commonly included in-class observations [33, 34, 36, 40], researcher-developed questionnaires [34, 36] and session reports [32, 34, 35].

**Table 3:** Evaluation frameworks of included studies

First author (Year published) Study	Evaluator	Indicators	Pre-Asses.	Process/ Implmt.	Post-Asses.	Follow Up	Tools (See Appendix)
Malti*.* (2008) [36] RALLY	Study Researchers	Development, resilience techniques, symptoms, relationships Program implementation	✓	✓	✓		SRM-SF; Researcher-developed resilience scale; YSR
Sibinga (2016) [30] MBSR	Program Staff	Mindfulness, psychological symptoms, anxiety, mood and emotion regulation, coping	✓		✓		CDI-S; SCL-90-R; MASC; PANAS; DES; STAXI-2; CRSQ; CSE
Kraag (2009) [39] Learn Young, Learn Fair	Maastricht University students	Stress management, coping, anxiety, depression	✓		✓	✓	STAIC; DIC-SF; MUSIC; SPSI
Mishara (2006) [32] Zippy’s Friends	Independent researchers	Student engagement, mood, behavior and emotion regulation, coping skills Program implementation	✓	✓	✓		Session reports; interviews; Social Skills Questionnaire; SSQTF; Schoolagers Coping Strategies Inventory; SSQSF
Clarke (2014) [33] Zippy’s Friends	Researcher & Health Promotion Specialist	Social and emotional literacy, social and emotional behavior Program implementation	✓	✓	✓	✓	Emotional Literacy Checklist; SDQ; Program Fidelity Checklist
Dufour (2011) [34] Zippy’s Friends	Undergraduate university students	Coping mechanisms, socio-emotional functioning, perceived social support, classroom climate Program implementation	✓	✓	✓		Observations; Session reports; Schoolagers Coping Strategy Inventory; Surveys; Socio-Emotional Profile; Social Support Scale for Children; Class Environment Climate Questionnaire
Holen (2012) [35] Zippy’s Friends	Teachers & Study Researcher	Coping skills	✓		✓		KidCope Questionnaire; SDQ
Clarke (2015) [31] Zippy’s Friends	Study Researcher	Coping skills, emotional literacy Program implementation	✓	✓	✓		Participatory workshop; draw and write technique; vignette response feelings activity; brainstorming
Nielsen (2015) [37] Up	Child and Adolescent Health Research Group at NIPH	Assertiveness, empathy, collaborative skills	✓	✓	✓	✓	Anonymous Surveys
Caldarella (2009) [40] Strong Start	Teachers & Research Assistants	Internalizing and externalizing behaviors, peer-related pro-social behavior Program implementation	✓	✓	✓		SSRS; Observations; Program fidelity checklist; IRP-15; Student Self-Assessment of Social Validity
Yamamoto (2017) [38] You Can Do It! Education	Study Researchers	Anxiety, Awareness of Social Support, Resilience	✓		✓		Spence children’s anxiety scale (SCAS), Social support scale for children (SSSC), Resilience in elementary school children scale (RESC)

*assess* assessment, *implmt* implementation

### Outcomes

Each article identified outcomes associated with their research question and hypothesis with outcomes following program implementation to assess the impact of the program. Table 4 presents a summary of whether major outcomes were considered by the article to have changed as a result of programing. In eight studies, researchers identified at baseline an overarching need for resilience programing among students, including low levels of trust and empathy; problems with emotion control, relationships and help-seeking; or reported symptoms [30, 31, 33, 36–40]. Ten out of eleven studies reported positive outcomes with improvements in student resilience and protective factors, including frequency of use of coping skills, internalizing behaviors, and self-efficacy at post-assessment [30–34, 36–40]. Three studies identified shortcomings in outcomes despite positive results from the overall program implementation and outcomes. Kraag et al. (2009) identified a lack of follow up and social reinforcement for components taught in programing, with negative implications on long-term follow-up outcomes [39]. Clarke and colleagues (2014) showed limited effects on resilience itself, but highlighted a marked increase in self-awareness among students [33]. Variations in outcomes between informants was highlighted in Holen et al (2012) who did not determine that resilience itself was an outcome of the program [35].

**Table 4:** Outcomes tracked and reported by each included study

First author, (Publication Year) Intervention	Resilience & Coping			Academic & Learning Motivation			Emotion and Behavior Self-Regulation			Relationships & Behavior			Psychological & Emotional Symptoms			Empathy		
	+ chng	No chng	n/a	+ chng	No chng	n/a	+ chng	No chng	n/a	+ chng	No chng	n/a	+ chng	No chng	n/a	+ chng	No chng	n/a
Malti (2008) [36] **RALLY**	✓			✓			✓			✓				✓		✓		
Sibinga (2016) [30] **MBSR**	✓					✓	✓					✓			✓			✓
Kraag (2009) [39] **Learn Young, Learn Fai**r	✓					✓			✓		✓		✓					✓
Mishara (2006) [32] **Zippy’s Friends**	✓						✓					✓			✓	✓		
Clarke (2014) [33] **Zippy’s Friends**	✓			✓			✓			✓				✓			✓	
Dufour (2011) [34] **Zippy’s Friends**	✓					✓			✓	✓			✓					✓
Holen (2012) [35] **Zippy’s Friends**		✓				✓	✓					✓			✓			✓
Clarke (2015) [31] **Zippy’s Friends**	✓					✓			✓	✓					✓	✓		
Nielsen (2015) [37] **Up**	✓					✓		✓		✓						✓		
Caldarella (2009) [40] **Strong Start**	✓					✓			✓	✓					✓			✓
Yamamoto (2017) [38] **You Can Do It! Education**	✓					✓	✓			✓			✓					✓

(*+ chng* positive change reported, *no chng* no change reported, *n/a* outcome not tracked or not applicable)

## Discussion

This review examined the program criteria and outcome measures used in the implementation and evaluation of resilience-focused, universal, school-based mental health promotion programs. Eleven published studies based on seven different programs were identified and included.

### Characteristics of effective programs

Several characteristics of effective programing stood out. The involvement of teachers in the delivery of programs emerged as key. Numerous studies used teachers to deliver the program, a feature presented positively as providing the opportunity for adaptability of programing and more seamless implementation, if provided with programmatic support and training. For example, the Zippy’s Friends program uses teachers to deliver the content materials [33] and teachers reported receiving substantial, helpful program support by research and program staff.

In their review of factors of success for implementation, adaptation of programing was identified as a key component of implementation [38, 41]. Teachers of the Zippy’s Friends Program reported the ability to adapt, add and remove activities relating to thematic content based on student literacy, mood and timing, as one of the most important parts of program delivery [33]. This allowed the maintenance of high program fidelity while also involving students in the most effective way possible. Teachers are an important resource in the development of children’s resilience, as they already have rapport and an understanding of the students and are more likely to know their students lived experiences and current coping and help-seeking strategies. Yamamoto et al. credit their successful implementation of the YCDI! Program with the extensive edits to the curriculum to adapt it to Japanese culture and relationships [38].

The length of programing did not appear to impact on the number of outcomes achievable. The RALLY program ran for an entire school year and provided consistent resilience outcomes [36], while the Mindfulness-Based Stress Reduction program ran for only 12 weeks and showed positive resilience outcomes as well [30]. The YCDI! Program ran for a shorter period of time than most implementations of the program but still demonstrated significant results [38]. Importance was placed on the intensity of sessions and the content delivered, as opposed to the regularity. However, importantly, if follow up evaluations were conducted, they did not reveal that outcomes were maintained in the longer term after most programs. This suggests that program length may not alter the ongoing resiliency of students once the program ends.

### Emergent themes across studies

Although all eleven articles presented programs that aimed at fostering the resilience skills and protective factors of students, the specific skills and outcomes taught in each program differed. This is consistent with research highlighting the difficulty that exists in defining resilience and creating programs around the topic [1]. Not only is the definition difficult and variable between studies, but the criteria and skills that come with developing resilience differ as well. In the RALLY study, researchers targeted resilience, and the outcomes evaluated were empathy, trust of others, and emotional regulation skills [36]. On the other hand, the UP study targeted resilience through social and emotional competencies that allow students to engage and navigate daily life, social interactions and society [37]. Both programs aimed to foster social and emotional development by increasing resilience skills and protective factors, but were based on differences in terminology and theory. Evaluations of both programs determined they had a positive outcome on resilience in students despite these differences.

An effect noted by a number of studies included in this review was the “ceiling effect” since many of the students enrolled in universal-based programs have high baseline mental health and social and emotional competence [33]. Although individuals within the group might suffer from higher risk factors or mental illness, across the board students present with generally normal levels. As such, when the program is implemented, outcomes may be generated but will not be large as there is little room for change. This is not the case when providing targeted programs with students who all generally have much more room for change, given that they begin the program with lower scores at baseline. Despite the ceiling effect, research has shown that resilience-boosting programing benefits at-risk but are not specific for at-risk children. Additionally, properly identifying and screening target groups for targeted programing is often unsuccessful due to the complexities of mental health, and preventive approaches, such as universal resilience-boosting programing, are considered the most all-encompassing method [42]. As such, a program promoting resilience will support positive changes and growth in both groups of kids, although with more significant differences in the at-risk group.

### Characteristics and methodologies of evaluations

An element of the evaluations that emerged in many articles is the removed nature of evaluation when collecting data on children’s capacities. Many of the programs seek to foster resilience through the development of coping skills, and use scales or observations in order to measure outcomes. The *Learn Young, Learn Fair* program evaluated a positive effect on emotion-focused, adaptive coping skills using validated questionnaires and scales [39]. This approach is used in all the program evaluations, but does not leave room for lived experiences to be factored into the interpretation of outcomes. These traditional evaluation methodologies can be seen as researching on a topic, rather than researching for a cause or population, as they do not leave room for ambiguity or other factors.

Additionally, a couple of studies in this review used evaluation tools that did not take into account the views of children themselves. The researchers chose to interview and evaluate both teachers’ and the program deliverers’ perceptions and ratings, rather than interviewing or evaluating the children themselves. For example, Caldarella, Christensen et al. (2009) evaluate children’s outcomes through pre- and post-assessments of the teacher’s perceptions of her students, using validated assessment tools [40]. However, evaluations like this introduce an additional limitation to the outcome analysis, as they gather data through secondary sources with the program delivered to children for their benefit, but outcomes not gathered directly from the children. However, observational data is a key component of a program evaluation with many studies successfully using observations to ensure program fidelity and as part of process evaluations.

More insight around outcomes occurs when multiple evaluation tools and methods are used [43]. Clarke and colleagues (2015) evaluated the use of a participatory workshop determining children’s coping skills which used draw and write techniques that allowed children to share their feelings using their own words rather than those of researchers [44], as well as vignettes to eliminate interview processes [45]. Students from the intervention group were found to use more adaptive coping skills in their daily life, both in and out of the classroom than children in the control group [31]. These results were supported by the quantitative data collected on the larger student sample from which the participatory workshop subsample was drawn [33]. A clearer picture of children’s coping skills and experiences with the Zippy’s Friends program was gathered through the use of both qualitative and quantitative evaluation methodology. Additionally, children’s lived experiences and direct insights were gathered through the participatory workshop model, allowing for a greater breadth of understanding on the program’s efficiency.

#### Limitations of articles and evidence

Consideration must be given to the ethics and feasibility of implementing and evaluating programs for mental health promotion. Ethical concerns arise from providing a program that might be highly beneficial for a group of children, and not for another, essentially disadvantaging them. The ethics are further confounded by the lack of complete or stringent randomization described in the studies that include a control group. To avoid the dilemma of disadvantaging students, studies on success factors have highlighted that in many studies the control groups do not receive ‘no intervention’ [41]. For example, Sibinga et al. (2016) included an active control group. While the intervention group received the Mindfulness-Based Stress Reduction program being studied, the control group received Healthy Topics, a general health program to match the MBSR structure. Thus, while the control group students are not receiving a resilience-focused, mental health promotion program, they still receive a health promotion program but one which allows a distinction between control and intervention groups around resilience outcomes and mental health [30]. Yamamoto and colleagues, however, did not provide programing to the control group following the intervention [38].

The evidence provided by certain articles must be weighed with differing criteria. Seven articles evaluated a program against a control group, allowing for comparison of outcomes. These articles present more substantial outcome evidence than those that do not include a control group for comparison. For example, Nielsen and colleagues (2015) and Caldarella and colleagues (2009) did not have a control group, decreasing the strength of their evaluation. Nielsen et al. (2015) implemented the UP program in kindergarten through grade 9, but only evaluated grades 5–9. Such selective evaluation introduces potential bias and paired with the absence of a control group makes it difficult to identify if the increase in social and emotional competencies is due to the UP intervention, or simply a natural developmental progression [37].

A limitation of the evaluations in many programs is the involvement of the person delivering the program as the evaluator. This can be seen in many studies on the Zippy’s Friends program, where the classroom teacher delivers the program and conducts the process and implementation evaluation themselves. Third-party observations are sometimes conducted in addition to verify program fidelity and implementation outcomes. Of note is that observational evaluation and the use of independent evaluators have been more extensively documented as reliable than using tools based on self-report [41].

We also note that despite gender differences in the prevalence of mental health problems and the type of resilience protective factors that children and adolescents use, the studies did not generally report results by gender [46, 47]. This limitation could be overcome by encouraging that future studies provide a gender breakdown or highlight gender-specific results.

## Conclusion

This review complements previous reviews on mental health promotion programing for students. Our focus on universally delivered programs in primary schools reveals key components and strengths of programing that make for the most successful delivery and evaluation and enables important conclusions to be drawn.

The review confirms that adaptability and teacher involvement are key elements of program delivery, with student engagement and use of multiple methods strengthening program evaluation. The use of participatory methods to engage children allows for greater assessment of lived experiences and use of coping skills compared to self-reporting tools or observations. Adaptability of curriculum to different contexts, seen in the *Zippy’s Friends* program, was considered successful by multiple authors, illustrating that broad program application across multiple contexts is possible and effective.

This review demonstrates the importance of establishing key criteria to be measured during delivery and evaluation of youth mental health promotion programs, particularly in terms of defining resilience and its associated indicators. The successes of the programs detailed by the studies included in this review highlight the need for and benefits of such programs. Further research on primary-school, universally delivered mental health promotion programs could be conducted in specific contexts, particularly more difficult settings such as developing countries or conflict zones.

## Appendix

**Table 5:** Evaluation tools and methodologies used in included studies

Criteria	Tool	First author (Year) of Studies in which Tool was Used	Purpose	Methodology	Timeframe
Depression	Children’s Depression Inventory, Short or Complete Form CDI	Sibinga (2016) [30] Kraag (2009) [39]	Assess depressive symptoms	Self-reported survey	Pre- assessment Post-assessment Follow up
Anxiety	Multidimensional Anxiety Scale for Children MASC	Sibinga (2016) [30]	Assess anxiety symptoms	Self-reported survey	Pre-assessment Post-assessment
	Spence Children’s Anxiety Scale SCAS	Yamamoto (2017) [38]	Assess frequency of anxiety symptoms	Self-reported survey	Pre-assessment Post-assessment
	Spielberger’s State-Trait Anxiety Inventory for Children STAIC	Kraag (2009) [39]	Assess anxiety symptoms	Self-reported survey	Pre-assessment Post-assessment Follow up
Social/Cognitive Development	Socio-moral Reflection Measure, Short Form SRM-SF	Malti (2008) [36]	Assess developmental levels of cognitive-moral skills	Paper and pencil self-reported survey	Pre-assessment Post-assessment
Resilience	Researcher-developed Resilience Scale	Malti (2008) [36]	Measure selected basic resilience factors, emotional regulation skills, academic skills	Self-reported survey	Pre-assessment Post-Assessment
	Resilience in Elementary School Children Scale RESC	Yamamoto (2017) [38]	Measure 19 elements relating to aspects of resilience	Self-reported	Pre-assessment Post-assessment
	Socio-Emotional Profile (Dumas et al, 1997)	Dufour (2011) [34]	Measure social competencies and adaption problems	80 items on six point scale, self-reported survey	Pre-assessment Post-assessment
Mindfulness	Children’s Acceptance and Mindfulness Measure	Sibinga (2016) [30]	Measure of mindfulness	10 item, self-reported survey	Pre-assessment Post-assessment
Symptoms	Youth Self Report YSR	Malti (2008) [36]	Assess behavioral and emotional functioning and symptoms	Self-reported survey	Pre-assessment Post-assessment
	Symptoms Checklist 90-R SCL-90-R	Sibinga (2016) [30]	Assess paranoid ideation, hostility, somatization	Self-reported survey	Pre-assessment Post-assessment
Stress	Children’s Post-Traumatic Symptom Severity Checklist CPSS	Sibinga (2016) [30]	Assess stress symptom severity and frequency	Self-reported survey	Pre-assessment Post-assessment
	Maastricht University Stress Instrument for Children MUSIC	Kraag (2009) [39]	Assess stress symptom severity and frequency	Self-reported scale survey Developed for study	Pre-assessment Post-assessment Followup
Relationships	Social Support Scale for Children	Dufour (2011) [34] Yamamoto (2017) [38]	Measure perceived social support by children: social support from parents, teachers, peers in class and intimate friends	24 items on four point scale	Pre-assessment Post-assessment
	School Social Behavior Skills SSBS	Caldarella (2009) [40]	Evaluate social competence and antisocial behavior of children	Norm-referenced, self-reported survey	Pre-assessment Post-assessment
	Social Skills Rating System SSRS	Caldarella (2009) [40]	Evaluate pro-social skills and problem behaviors of students	Six-subscale items, norm-referenced, self-reported survey	Pre-assessment Post-assessment
	Social Skills Questionnaire Teacher Form, Elementary Level SSQTF Student Form, Elementary Level SSQSF	Mishara (2006) [32]	Measure frequency of observed behaviors and social skills; scores determined for cooperation, assertion, self-control	Rating of frequency of specific behaviors	Process evaluation
Mood & Emotions	Positive and Negative Affect Schedule PANAS	Sibinga (2016) [30]	Assess mood and emotion regulation	Self-reported survey	Pre-assessment Post-assessment
	Differential Emotional Scale DES	Sibinga (2016) [30]	Assess mood and emotion regulation	Self-reported survey	Pre-assessment Post-assessment
	Aggression Scale	Sibinga (2016) [30]	Assess mood and emotion regulation	Self-reported survey	Pre-assessment Post-assessment
	State-Trait Anger Expression Inventory STAXI-2	Sibinga (2016) [30]	Assess mood and emotion regulation	Self-reported survey	Pre-assessment Post-assessment
	Strengths and Difficulties Questionnaires SDQ	Clarke (2014) [33] Holen (2012) [35]	Assess children’s emotional and behavioral functioning: emotional symptoms, conduct 2problems, hyperactivity, peer relationship problems, pro-social behavior	Self-reported questionnaire, 25 items with five main subscale scores	Pre-assessment Post-assessment Follow up
	Feelings Activity	Clarke (2015) [31]	Assess ability to identify feelings in response to problem situations	Part of participatory workshop evaluation: 6 vignettes read aloud and children asked to respond and explain each	Process evaluation Post-assessment
	Social and Emotional Competence Index	Nielsen (2015) [37]	Assess assertiveness, empathy and collaborative skills	Self-reported questionnaire with ranked answers	Pre-assessment Post-assessment
	Emotional Literacy Checklist	Clarke (2014) [33]	Assess emotional literacy: self-awareness, self-regulation, motivation, empathy and social skills	Self-reported questionnaire, 20 items rated on four-point Likert Scale	Pre-assessment Post-assessment Follow up
Coping	Children’s Response Style Questionnaire CRSQ	Sibinga (2016) [30]	Assess coping ability by measuring 3 types of reactions: rumination, problem solving, destruction	Self-reported survey	Pre-assessment Post-assessment
	Brief COPE	Sibinga (2016) [30]	Assess coping ability by measuring 14 coping approaches	Self-reported survey	Pre-assessment Post-assessment
	Coping Self-Efficacy Scale CSE	Sibinga (2016) [30]	Assess coping ability	Self-reported survey with end result of single variable	Pre-assessment Post-assessment
	Social Problem-Solving Inventory SPSI	Kraag (2009) [39]	Measure problem-solving skills	Self-reported scales	Pre-assessment Post-assessment Follow up
	Schoolagers Coping Strategies Inventory	Mishara (2006) [32] Dufour (2011) [34]	Gather information about actual coping experiences of children; identify frequencies of use of coping skills	Self-reported questionnaire, 26 items	Process evaluation Post-assessment
	Draw and Write Technique	Clarke (2015) [31]	Gather personal experiences of how children coped with problem situations	Part of participatory workshop: children asked to draw picture about emotion and draw how they coped	Pre-assessment Post-assessment
	KidCope Questionnaire	Holen (2012) [35]	Measure of 10 coping strategies: distraction, social withdrawal, cognitive restructuring, self-criticism, blaming others, problem solving, emotional expression, wishful thinking, social support, resignation	Self-reporting questionnaires with adaptations for different age groups	Pre-assessment Post-assessment
	Focus Groups	Malti (2008) [36]	Evaluation of program implementation and process	Multiple students with one researcher	Process evaluation Implementation evaluation Post-assessment
	Researcher-developed questionnaires	Malti (2008) [36] Dufour (2011) [34]	Assessment of components of program; assessment of children’s status by parents and teachers	Self-reported questionnaires	Pre-assessment Process evaluation Post-assessment Climate assessment
	Session Reports	Mishara (2006) [32] Dufour (2011) [34] Holen (2012) [35]	Assessment of each component of program	Qualitative, self-administered report by program deliverer after each session	Process evaluation Implementation evaluation
	Interviews	Malti (2008) [36] Clarke (2015) [31]	Assessment of components of program	One-on-one interviews with researcher or evaluator	Pre-assessment Process evaluation Post-assessment Implementation evaluation
	Programme Fidelity Checklist	Clarke (2014) [33] Caldarella (2009) [39]	Report of what portions of program session fully or partially implemented, which ones omitted	Self-reported checklist and questionnaire	Process assessment Implementation evaluation
	Class Environment Climate Questionnaire	Dufour (2011) [34]	Assess climate of classroom and describe teacher characteristics	Self-reported questionnaire, 36 items	Pre-assessment Post-assessment
Acceptability	Student Self-Assessment of Social Validity	Caldarella (2009) [40]	Assess student perception of social validity of program	Self-reported questionnaire, 10 questions: 8 with Likert Scale, 2 open ended	Post-assessment
	Intervention Rating Profile-15 IRP-15	Caldarella (2009) [40]	Assess teacher’s perception of social validity of program	Self-reported questionnaire with 15 items on 6-point Likert scale	Post-assessment

## Abbreviations

AOP-PTS: Aussie optimism: positive thinking skills program
CDI: Children’s depression index
MBSR: Mindfulness-Based stress reduction
MMAT: Mixed methods appraisal tool
WA: Western Australia
